# Isolation of Vaginal Epithelial Cells: In Preparation of Autologous Vaginal Tissue Lining for Congenital Absence of Vagina Patients

**DOI:** 10.3390/ijms24108798

**Published:** 2023-05-15

**Authors:** Too Lih Yuan, Nadiah Sulaiman, Abdul Ghani Nur Azurah, Manira Maarof, Rabiatul Adawiyah Razali, Benson Koh, Roszita Ibrahim, Ani Amelia Zainuddin, Muhammad Dain Yazid

**Affiliations:** 1 Centre for Tissue Engineering & Regenerative Medicine, Faculty of Medicine, Universiti Kebangsaan Malaysia Medical Centre, Cheras, Kuala Lumpur 56000, Malaysia; lihyuan@outlook.com (T.L.Y.); nadiahsulaiman@ukm.edu.my (N.S.); manira@ppukm.ukm.edu.my (M.M.); rabiatularzl@ukm.edu.my (R.A.R.); benson9584@gmail.com (B.K.); 2 Department of Obstetrics and Gynecology, Faculty of Medicine, Universiti Kebangsaan Malaysia Medical Centre, Cheras, Kuala Lumpur 56000, Malaysia; nurazurahag@gmail.com (A.G.N.A.); aniamelia@ukm.edu.my (A.A.Z.); 3 Department of Public Health, Faculty of Medicine, Universiti Kebangsaan Malaysia Medical Centre, Cheras, Kuala Lumpur 56000, Malaysia; roszita@ppukm.ukm.edu.my

**Keywords:** congenital absence of the vagina, vaginal epithelial cells, cell isolation, epithelial–mesenchymal transition, mesenchymal–epithelial transition

## Abstract

Infertility is a condition affecting women who are born with an underdeveloped or absent vagina, a birth defect known as congenital absence of the vagina. It is a rare disorder where the development of the Mullerian duct is obstructed by unidentified causes. The case is seldom reported due to the low prevalence and sparse epidemiology studies worldwide. A potential solution for the disorder is neovaginal creation with in vitro cultured vaginal mucosa. Limited studies have reported its application, but none are reproducible or specific regarding the established processes for acquiring vaginal epithelial cells from vaginal biopsies. These research gaps were adequately answered with an epidemiology study of inpatient details in Hospital Canselor Tuanku Muhriz, Malaysia, established methods and outcomes of vaginal tissue processing and isolation, and characterization of vaginal epithelial cells using 3-(4,5-dimethylthiazol-2-yl)-2,5-diphenyl-2H-tetrazolium bromide (MTT) and immunofluorescence assays. The reported evidence and speculation that the disorder arises because of a cellular transition event between epithelial and mesenchymal cells during the development of the Mullerian duct could be key in the creation of neovaginas using established culture procedures to improve surgical results and restore fertility.

## 1. Introduction

Congenital absence of the vagina (CAV) is an atypical condition where unexpectedly the vagina does not develop before birth. It is a Mullerian anomaly that emerges following a disruption at a certain stage of embryonic development, and sometimes affects non-reproductive systems as well [1]. The infertility accompanied by CAV often causes an impact on the physical and psychosocial welfare of women. The low prevalence and limited epidemiological studies nationwide restrict the understanding of this disorder. Accomplished CAV studies aimed at neo-vaginoplasty with diverse surgical approaches and neovaginal materials are relatively case-specific due to symptom variations [2]. Recent studies have applied in vitro cultured vaginal mucosa, a novel and promising neovaginal graft, but the details on vaginal tissue processing, cell isolation methods and vaginal epithelial cell culture were yet to be established [3,4]. This study aimed to demonstrate the necessity and outcomes of an established vaginal epithelial cell isolation method in preparation for the reconstruction of vaginas. This is the primary study that reports on Malaysian prevalence of CAV collected from a single center, i.e., Hospital Canselor Tuanku Muhriz (HCTM), in addition to describing the methods and outcomes of tissue processing, vaginal cell isolation, vaginal epithelial cell features and reviewing the cause of CAV. The findings could greatly benefit future advances in vaginal epithelialization, and the successful establishment of in vitro cultured vaginal mucosa would be one of the important stages in restoring female infertility.

## 2. Results and Discussion

### 2.1. Characteristics of Patients with CAV

**Figure 1:**
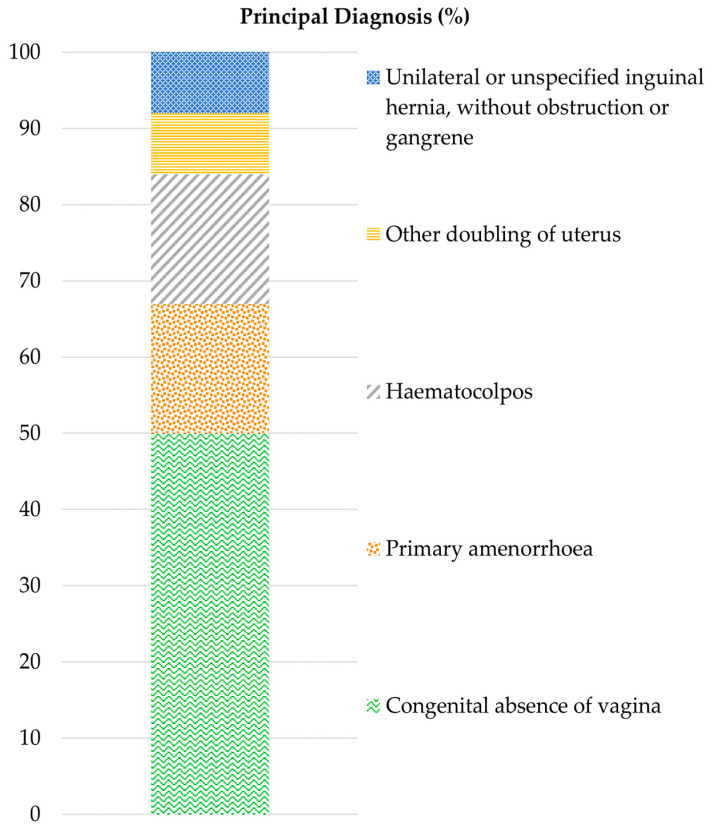
Diagnostic outcomes and management of patients with congenital absence of the vagina in Hospital Canselor Tuanku Muhriz, Malaysia for the past 10 years.

During the 10-year period, only 11 patients were reported in HCTM, Malaysia, with the age range between 12 and 34 years old. CAV was the main diagnosis (50.0%), followed by primary amenorrhea (17.0%), hematocolpos (17.0%) and doubled uterus or inguinal hernia (8.0%) (Figure 1). There were no records for patients below the age of puberty as most cases were only observed when they missed menstrual periods and had pelvic or abdominal pain. These patients showed normal development of female secondary sexual characteristics, which misleads them for having a complete female reproductive system.

**Figure 2:**
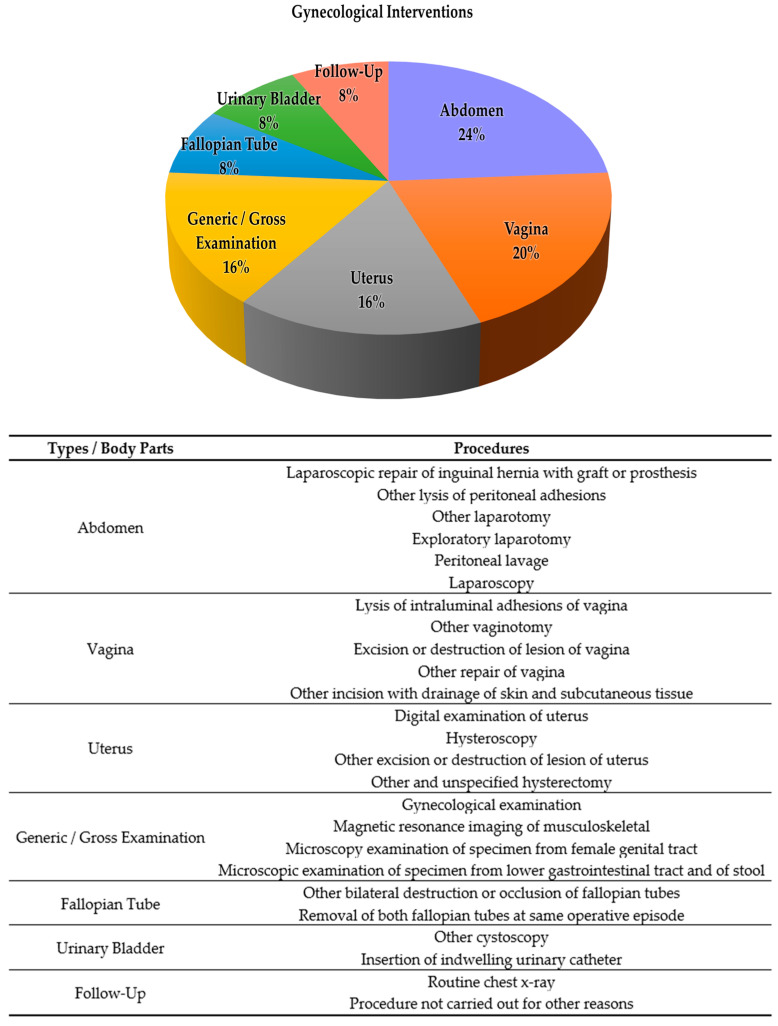
Tabulation of gynecological interventions conducted on patients with congenital absence of the vagina.

To date, there is no standard procedure or cure for CAV as it is an inborn and rare abnormality. CAV cases in HCTM are often emergencies, where patients arrive in pain with diverse clinical presentations. Multiple gynecological interventions were conducted on these patients, mostly interventions on the abdomen (24.0%), with interventions on the vagina being either for examination purposes or as a symptom remedy (20.0%). Other interventions were associated with the uterus and generic examination (16.0%) as well as interventions on fallopian tubes, bladder and follow-up sessions (8.0%) (Figure 2). Ultimately, the current treatments conducted on patients with CAV are symptom dependent. Similarly, Minami et al. [5] reported on two cases of congenital vaginal agenesis with hematometra and lower abdominal pain. Pelvic magnetic resonance imaging (MRI) of the first patient showed clotted blood filling the uterine cavity and drainage was conducted to relieve lower abdominal pain, while the pelvic MRI results of the second patient revealed an 8 cm-sized chocolate cyst in the left ovary, which was removed via left ovarian cystectomy. In another case reported by Oliveira and Ferreira [6], an 18-year-old patient diagnosed with uterine and vaginal agenesis underwent a mini-laparoscopic modified Vecchietti procedure for neovaginal creation.

A two-step procedure of vaginal intervention entails the creation of a neovagina and later anastomosis of the uterus with the newly created vagina, which requires the establishment of an autologous vaginal tissue lining. Since research into vaginal epithelial cell isolation methods is extremely limited, this study aimed to establish a proper isolation method illustrating the morphological descriptions of in vitro vaginal epithelial cells (VECs) from patients who underwent vaginal septotomy.

### 2.2. Collagenase Demonstrated Effective Tissue Digestion for Primary VEC Isolation

**Figure 3:**
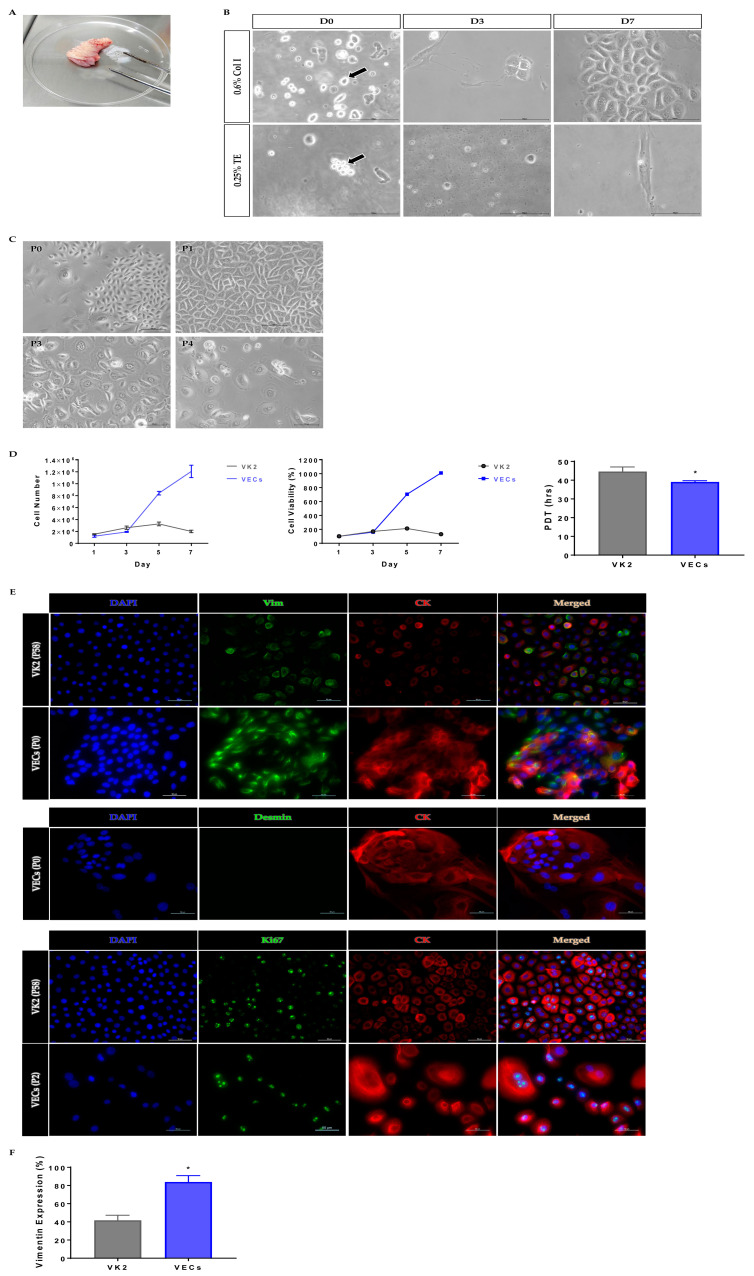
(**A**) Vaginal tissue biopsy. (**B**) Morphological observation of vaginal epithelial cells after digested with 0.6% collagenase type I enzyme (arrow depicts single, well-isolated cells) and 0.25% trypsin-EDTA (arrow depicts clumped cells). Scale bar = 100 µm. (**C**) Morphological observation of vaginal epithelial cells across passages. Scale bar = 100 µm. (**D**) Cell growth analyses (* *p* < 0.05). (**E**) Immunofluorescence analysis. Scale bar = 50 µm. (**F**) Comparison of vimentin expression (* *p* < 0.05). D: day; P: passage number; PDT: population doubling time; Vim: vimentin; CK: pan-cytokeratin [AE1/AE3 + 5D3]; DAPI: 4′,6-diamidino-2-phenylindole; Ki67: nuclear antigen Ki67.

An amount of 0.6% collagenase type I enzyme (Col I) and 0.25% trypsin-EDTA (TE) were utilized to digest vaginal tissues (Figure 3A). VECs were successfully isolated and found at a high yield when digested using Col I compared to TE, where the cells were mostly found clumped. On day 3, Col I-digested cultures showed complete attachment compared to TE-digested cultures, where the cells remained spherical in shape and free-floating. On day 7, Col I-digested VEC numbers were increased with retained epithelial-like morphology. Meanwhile, TE-digested, attached VECs became elongated and lost their epithelial-like morphology, presumably becoming fibroblastic (Figure 3B). Previous studies had proven that collagenase yielded a high number of cells, gave satisfactory cell attachment [7] and improved cell surface protein preservation [8], while trypsin was found to be harsh to cells, which resulted in lower cell yield [9] and alterations to cell membrane proteins [10] and led to cell lysis [11]. This demonstrated that 0.6% Col I is a suitable dissociation enzyme for VEC isolation from vaginal tissue biopsies.

### 2.3. Primary VECs Revealed Best Epithelial Morphology at Early Passage

VECs were passaged up to P4 with gradual loss of polygonal epithelial morphology, as the passage number increased (Figure 3C). This might be due to the occurrence of an irreversible differentiation process, where the elongation and enlargement of cell shape [12,13,14] were seen. Further, P1 culture showed the best epithelial morphology compared to P3 and P4 cultures, which indicates that early passage number VECs are preferably used to construct neovaginas, as the cells deteriorate with increasing passage number [15].

### 2.4. Primary VECs Exhibited High Proliferation Rate

VEC number showed an increase up to day 7 of culture, whilst VK2 cell number decreased after day 5 of culture. The percentage of cell viability for both cells also showed a similar trend. VECs demonstrated a higher percentage of viable cells, even on the last day of analysis, compared to VK2. Proliferation analysis showed that the population doubling time (PDT) of VECs (39.05 ± 0.66 h) was significantly shorter than VK2 (44.61 ± 2.45 h), suggesting VECs require shorter times to increase two fold in the population (*p* < 0.05). These data (Figure 3D) suggest that our monolayer culture technique produces better outcomes in terms of cell yield, cell viability and proliferation rate when compared to the time-consuming explant method [4].

### 2.5. Primary VECs Exhibited High Vimentin Expression

Immunofluorescence analysis was carried out to identify the types of proteins present in the VEC population isolated from vaginal tissue (Figure 3E). Primary VECs exhibited significantly higher vimentin expression of 83.8 ± 0.07% compared to VK2 cells (41.6 ± 0.06%) (*p* < 0.05) (Figure 3F). Though vimentin is a major epithelial cell intermediate filament [16], the high vimentin expression in epithelial cells indicates that epithelial–mesenchymal transition (EMT) occurs within the VEC population. Sivagurunathan et al. [17] found that induction of vimentin expression in MCF7 epithelial cells upregulated TWIST1 (specific transcription factor of EMT), CDH11, MMP16 and MME (mesenchymal markers), exhibiting EMT. Another study showed that induction of vimentin in MCF7 cells caused the epithelial cells to adopt a mesenchymal shape and increase in motility, resembling features of the EMT [18]. EMT is induced during the development of the Mullerian duct for epithelializing vagina [19], epithelial cells change their morphology and biochemical properties to mesenchymal-like via pathways such as the Wnt/β-catenin signaling pathway, and the epithelialization process completes with the induction of the mesenchymal–epithelial transition (MET) by regulatory genes [20,21]. The dysregulation of EMT may result in partial or complete absence of the vaginal structure [22].

Both VK2 and VECs stained positive for Ki67, indicating cell proliferation. The expression of desmin was determined to validate the presence of muscle cells in the epithelial population. As no desmin was expressed in the population, this suggests that the isolation method could successfully isolate the homogenous culture of vaginal epithelial cells with the absence of skeletal and smooth muscle cells [23], which are commonly unwanted populations in neovaginal construction. 

## 3. Materials and Methods

### 3.1. Ethical Approval

The study was approved by the Research Ethics Committee of UKM (UKM PPI/111/8/JEP-2021-519) and informed written consent was obtained from patients before enrolment.

### 3.2. Inpatients with CAV in HCTM, Malaysia

To identify the number of patients with CAV and explore the diagnostic and procedural information associated with these patients, inpatient data of 11 individuals with CAV were obtained from the Casemix system developed by the International Centre for Casemix and Clinical Coding (ITCC), HCTM. The diagnosis and procedure details were retrieved according to the International Statistical Classification of Diseases and Related Health Problems, 10th Revision (ICD code: Q52.0; congenital absence of vagina) on 7 December 2022. The search was refined to a 10-year period (2012–2022) of inpatients with CAV admitted to HCTM, Malaysia. The data were analyzed with Microsoft Excel (version 2304), presenting the characteristics of the patients.

### 3.3. Vaginal Tissue Processing and Cell Isolation

To establish a vaginal epithelial cell isolation method, vaginal tissue was obtained with consent from three patients who had undergone vaginal septotomy. The tissue was transported to the Centre for Tissue Engineering and Regenerative Medicine (CTERM), the National University of Malaysia (UKM) in sterile containers containing saline solution. At least 1 cm^2^ of full-thickness vaginal biopsies were required and processed aseptically within 24 h. The tissue was placed into a sterile Petri dish and cleaned of blood, fats, sutures and undesired substances. The tissue was rinsed with sterile phosphate-buffered saline (Sigma-Aldrich, St. Louis, MO, USA) containing 1.0% of Antibiotic/Antimycotic Solution (Capricorn Scientific, Ebsdorfergrund, Germany). To evaluate the effectiveness of different tissue dissociation enzymes in isolating VECs, the tissue was then minced and incubated either with 0.6% Col I (Worthington Biochemical Corporation, Lakewood, NJ, USA) or 0.25% TE (Capricorn Scientific, Ebsdorfergrund, Germany) in an incubator shaker at 37 °C, 250 rpm for 1.5 to 2 h for tissue dissociation. For the Col I incubation procedure, the cells were pelleted and re-incubated with 0.05% TE for an additional 8–10 min and vortexed vigorously. After trypsin inhibition, the cells were washed and resuspended with media before being seeded onto 6-well plates for further expansion. 

### 3.4. Cell Cultures

Primary vaginal cultures were first established in a 1:1 ratio of DMEM/F-12 medium (American Type Culture Collection, Manassas, VA, USA) supplemented with 10.0% fetal bovine serum (Capricorn Scientific, Ebsdorfergrund, Germany): Epilife medium with Human Keratinocyte Growth Supplement (Gibco, San Diego, CA, USA) in a 37 °C, 5.0% CO_2_ incubator. After initial seeding, the cells were monitored every 2 to 3 days for cell attachment and morphology. Once the cells had reached approximately 80% confluence, differential trypsinization was carried out to remove fibroblasts from the culture. This step was done by washing the culture with sterile phosphate-buffered saline containing 1.0% of Antibiotic/Antimycotic Solution, treating it with TrypLE Select (Gibco, San Diego, CA, USA) for at most 3 min, removing supernatant containing detached fibroblasts and adding complete Epilife medium into the well containing adherent vaginal cells. The vaginal cells continued to culture in complete Epilife medium up to passage 4 with the medium being changed every 2 to 3 days.

A vaginal cell line VK2/E6E7 (ATCC CRL-2616), obtained from ATCC (American Type Culture Collection, Manassas, VA, USA), was used as a control. The cell line was revived according to the manufacturer’s instructions and cultured with the recommended medium: Keratinocyte serum-free medium supplemented with 0.05 mg/mL Bovine Pituitary Extract and 0.1 ng/mL human recombinant Epidermal Growth Factor (Gibco, San Diego, CA, USA). The cultures were maintained at 37 °C in a 5.0% CO_2_ incubator with the medium changed every 2 to 3 days. 

Cells were seeded at a density of 1 × 10^4^/cm^2^ for further analyses.

### 3.5. Analysis of Cell Proliferation and Viability

For assessing the cell growth rate, 3-(4,5-dimethylthiazol-2-yl)-2,5-diphenyl-2H-tetrazolium bromide (MTT) assay was performed in triplicate in accordance with the manufacturer’s instructions on day 1, 3, 5 and 7. Briefly, the MTT reagent (Thermo Fisher Scientific, Waltham, MA, USA) was added into each well and incubated for 3 h at 37 °C in the dark. Dimethyl sulfoxide (Sigma-Aldrich, St. Louis, MO, USA) was then added into each well and the absorbance of dissolved formazan was measured at 540 nm. MTT assay was also performed on a series of known cell numbers to generate a standard curve (cell number versus absorbance) for quantifying the cell number and viability.

The calculation of PDT was carried out as described by Miyazawa et al. [24], according to the following equation: PDT = *t*Log2/(Log*N2* − Log*N1*), where *t* denotes time in culture (in hours), *N2* denotes the cell number at the end of the passage and *N1* denotes the cell number seeded at the beginning of the passage.

### 3.6. Immunofluorescence Analysis

To evaluate the types of proteins expressed in the vaginal cells, the cells were fixed with 4.0% of paraformaldehyde (Merck, Rahway, NJ, USA) for 15 min, permeabilized with 0.5% Triton X-100 (Sigma-Aldrich, St. Louis, MO, USA) for 10 min, blocked with 10.0% goat serum (Sigma-Aldrich, St. Louis, MO, USA) at 37 °C for 1 h, stained with recombinant anti-pan cytokeratin [AE1/AE3 + 5D3]—AlexaFluor^®^ 594 anti-mouse, anti-Ki67 (Abcam, Cambridge, UK), anti-desmin (D93F5) and anti-vimentin (D21H3) (Cell Signalling Technology, Danvers, MA, USA)—Alexa Fluor^®^ 488 anti-rabbit (Abcam, Cambridge, UK) and finally completed with a 40 min DAPI (Invitrogen, Waltham, MA, USA) staining. Images were captured using a Nikon Eclipse Ti Fluorescence microscope (Nikon, Tokyo, Japan).

### 3.7. Statistical Analysis

Statistical analysis was performed with ANOVA using GraphPad Prism 7.04 with all values expressed as mean ± SD and statistical significance considered at *p* < 0.05.

## 4. Conclusions

In summary, various clinical manifestations of patients from HCTM, Malaysia were recorded, with CAV being the most reported diagnosis, followed by primary amenorrhea and haematocolpos. Treatment options are dependent on the symptoms displayed. The protocol establishment of epithelial cell isolation from post-surgery redundant vaginal tissue can be seen as a feasible solution for the fabrication of autologous neovaginas. Since the isolated VECs exhibited EMT condition, which potentially causes vaginal obstruction recurrence leading to infertility, an intervention such as gene manipulation is imperative to induce MET in patient cells before a new vaginal lining is established. This study could serve as a basis for future epithelial tissue lining establishment to create autologous neovaginas. The development of neovaginal grafts not only relieves the conditions such as hematometra, but also potentially addresses the conception and delivery difficulties of women with infertility. 

## Data Availability

Not applicable.
